# Comparative Efficacy of Phacoemulsification Combined with KDB Goniotomy versus Trabeculectomy for Primary Angle-Closure Glaucoma: A Retrospective Cohort Study

**DOI:** 10.7150/ijms.125193

**Published:** 2026-02-11

**Authors:** Xu Hou, Jing Wu, Xiaxia Yang, Jian Zhou, Dan Hu

**Affiliations:** Eye Institute of Chinese PLA and Department of Ophthalmology, Xijing Hospital, Fourth Military Medical University, Xi'an, Shaanxi 710032, China.

**Keywords:** minimally invasive glaucoma surgery, goniotomy, trabeculectomy, phacoemulsification, primary angle closure glaucoma

## Abstract

**Objective:**

This study compared the efficacy and safety between phacotrabeculectomy (phaco-trab) and phacoemulsification combined with Kahook Dual Blade goniotomy (phaco-KDB) in patients with moderate to severe primary angle-closure glaucoma (PACG).

**Methods:**

This single-center, non-randomized retrospective cohort study included 77 eyes from 74 PACG patients with concurrent cataract, who underwent either phaco-trab (40 eyes, 39 patients) or phaco-KDB (37 eyes, 35 patients). All patients had moderate-to-severe visual field (VF) defects and were using ≥ 2 topical intraocular pressure (IOP)-lowering medications preoperatively. Changes in best-corrected visual acuity (BCVA), IOP, medication use, mean deviation (MD) progression of VF, and complications were compared between the two groups.

**Results:**

The comparable follow-up duration was 13.5 ± 4.1 months (phaco-trab) and 12.9 ± 3.9 months (phaco-KDB) (*P* = 0.703). Postoperative BCVA improved in both groups, with no significant intergroup difference. The baseline IOP was 30.29 ± 7.69 mmHg in the phaco-trab group and 28.20 ± 4.70 mmHg in the phaco-KDB group, which decreased to 15.64 ± 3.22 mmHg and 17.55 ± 3.34 mmHg respectively (*P* = 0.041), and the phaco-KDB group required more medications (*P* =0.027) at the last follow-up. The qualified success rates were 87.5% (phaco-trab) versus 83.8% (phaco-KDB) (*P* = 0.604). No statistically significant progression of VF defects and no serious intraoperative or postoperative complications were noted.

**Conclusions:**

Both KDB goniotomy and trabeculectomy, when combined with phacoemulsification, effectively improve visual acuity, reduce IOP, and stabilize VF in patients with moderate-to-severe PACG and concurrent cataract. Phaco-KDB is an effective *ab interno* procedure that achieves comparable success rate to phaco-trab, it's minimally invasive nature may delay or obviate filtration surgery, simplify procedure, and offer faster visual rehabilitation.

## Introduction

Glaucoma, the leading cause of irreversible blindness, requires intervention in nearly all cases, including topical pharmacotherapy, laser therapeutic modalities, or surgical procedures, to reduce intraocular pressure (IOP) and slow the progression of visual field deterioration.^1^ Projections indicate that by 2040, 111.8 million people globally will be affected by glaucoma.^2^ Primary angle-closure glaucoma (PACG) exhibits a significantly higher prevalence in East Asian populations compared to other geographic regions. In PACG, appositional or synechial closure of the iridocorneal angle obstructs aqueous humor outflow, leading to progressive IOP elevation, optic neuropathy with characteristic cupping of the optic disc, and corresponding visual field deficits. In the Chinese population, trabeculectomy remains the primary initial surgical intervention for moderate-to-severe PACG; clinical decisions are guided by parameters such as baseline IOP with tolerated eye drops, the extent of peripheral anterior synechiae (PAS), retinal nerve fiber layer (RNFL) thinning, and the degree of visual field compromise. 3 Trabeculectomy with or without concomitant cataract extraction has been established as the first-line treatment strategy for medically refractory PACG.^4^ Despite its efficacy, trabeculectomy carries inherent risks, including anterior chamber shallowing, hypotony, malignant glaucoma, and bleb-related complications; these risks require detailed preoperative discussion with patients.^5^

Over the past two decades, minimally invasive glaucoma surgery (MIGS), characterized by minimized tissue trauma and enhanced safety profiles, has experienced substantial adoption.^6^ Classified according to their mechanistic targets for IOP reduction, MIGS procedures can be categorized into those augmenting trabecular outflow, enhancing uveoscleral outflow, creating subconjunctival drainage pathways, or ablating ciliary body function.^7^ Performed via clear corneal incisions, MIGS interventions may be combined with phacoemulsification in the management of primary open-angle glaucoma (POAG) or PACG. Procedures such as goniotomy and canaloplasty have demonstrated increased utilization among US Medicare beneficiaries.^8^ In China, a notable trend has emerged in recent years: a decline in the performance of ab externo filtration surgeries and a corresponding increase in the adoption of *ab interno* drainage procedures. 9 Collectively, these observations highlight a global paradigm shift toward minimally invasive approaches in glaucoma surgery. Importantly, the therapeutic benefits of MIGS in moderate or severe PACG remain less well established compared to its role in POAG.

This clinical study aimed to evaluate the efficacy and safety of Kahook Dual Blade (KDB; New World Medical, Rancho Cucamonga, CA, USA) goniotomy combined with phacoemulsification (phaco-KDB) in patients with moderate-to-severe PACG and concurrent cataract. Primary outcome measures included best-corrected visual acuity (BCVA), IOP control, glaucoma medication burden, VF defect progression and complications; with comparisons made with the phaco-trab cohort.

## Methods

This single-center, non-randomized retrospective cohort study (KY20193168-1) was approved by the Institutional Ethics Committee and Academic Board of Xijing Hospital, Fourth Military Medical University, Xi'an, China. All procedures adhered to the ethical principles outlined in the World Medical Association Declaration of Helsinki. Written informed consent was obtained from all participants prior to their enrollment in the study.

Patients with PACG and concurrent cataract, who underwent phaco-KDB as initial surgical treatment at the Eye Center of Xijing Hospital between January 2022 and June 2024, or phaco-trab between January 2019 and December 2023, were included in this study. Clinical data collected included: BCVA (logMAR), IOP (Goldmann applanation tonometry), number of glaucoma medications, anterior chamber depth (ACD), extent of PAS, and visual field mean deviation (MD) (dB, Humphrey 30-2 SITA Standard perimetry). All enrolled patients exhibited: a prior laser peripheral iridotomy (LPI), clinically cataract, PAS (≥ 90°), moderate (MD: -6 dB to -12 dB) to severe (MD: < -12 dB) visual field loss, and ≥ 2 topical IOP-lowering medications, including combinations of: cholinergics (pilocarpine), beta-blockers (timolol), carbonic anhydrase inhibitors (brinzolamide), alpha-2 adrenergic agonists (brimonidine). Patients were excluded if they had: a history of prior ocular surgery, concurrent ocular diseases (age-related macular degeneration, diabetic retinopathy, or uveitis), or uncontrolled systemic diseases.

All operations were performed by a single glaucoma surgeon (X.H.) with standardized techniques. The surgical sequence was as follows: following topical anesthesia (proparacaine 0.5%) and sterile preparation, a 2.4-mm temporal clear corneal two-plane incision was created. A 5.0-5.5-mm continuous curvilinear capsulorhexis was performed, followed by phacoemulsification using the stop-chop technique with the Whitestar Signature phacoemulsification system (Johnson & Johnson, New Brunswick, NJ, USA). A three-piece hydrophobic acrylic intraocular lens (IOL) (PY-60AD, Hoya, Tokyo, Japan, or AR40E, Johnson & Johnson, Jacksonville, FL, USA) was implanted in the capsular bag, followed by viscoelastic removal with automated irrigation-aspiration. Intracameralcarbachol (1:1000) was administered to constrict the pupil. The iridocorneal angle was visualized using a VTSTVG surgical gonioprism (Volk Optical, Mentor, OH, USA). Under high magnification microscopy, viscoelastic and chop hook dissection was performed, circumferentially separating PAS to re-open the trabecular meshwork outflow pathway.

Goniotomy technique: following phacoemulsification and IOL implantation, the KDB device was introduced through the main corneal incision. Using its angled tip, the surgeon performed selective lysis of peripheral iridotrabecular adhesions (if present) to expose the trabecular meshwork (TM). Once the TM was adequately visualized, a 3-4 clock-hour incision of Schlemm's canal was performed using the dual-blade mechanism, extending from the anterior limit of the scleral spur to the posterior aspect of the TM. Complete removal of intraocular viscoelastic was achieved via balanced salt solution irrigation, followed by hydraulic wound closure of the clear corneal incision to ensure aqueous seal integrity. Trabeculectomy technique: the trabeculectomy procedure followed our established protocols 10.

Complete success: IOP ≤ 18 mmHg without medications, qualified success: IOP ≤18 mmHg with ≤ 2 topical medications. Failure criteria included: IOP > 21 mmHg despite the use of > 2 topical glaucoma medications; hypotony (IOP < 6 mmHg); requirement for additional glaucoma surgeries (trabeculectomy or tube shunt placement); MD progession of visual field ≥6 dB; and no light perception. Suture removal for IOP modulation, bleb needling, or postoperative laser procedure were not classified as failure.

Statistical analysis was conducted using IBM SPSS Statistics Version 26.0 (IBM Corp., Armonk, NY, USA). Continuous variables were reported as mean ± standard deviation (SD), while categorical data were presented as absolute numbers (n) and percentages (%). The Kolmogorov-Smirnov test was used to assess normality of data distribution. For parametrically distributed variables, one-way analysis of variance (ANOVA) with post-hoc Tukey tests was employed for intergroup comparisons. Non-parametric variables were analyzed using the Mann-Whitney U test. Surgical success rates were evaluated using Kaplan-Meier survival analysis with the log-rank test for between-group comparisons. All statistical tests were two-sided, and a *P* value < 0.05 was considered statistically significant.

## Results

### Patient Characteristics

Of the 74 PACG patients (77 eyes) recruited in the study, 39 patients (40 eyes) underwent phaco-trab and 35 age- and sex-matched patients (37 eyes) underwent phaco-KDB. Patient demographics and baseline ocular characteristics are summarized in Table 1. No statistically significant differences were observed between the two groups in terms of preoperative BCVA, IOP, glaucoma medications, or ocular parameters including ACD, axial length, lens thickness, PAS, VF defect, and IOL power. The mean follow-up duration was comparable between the two groups, with 13.5 ± 4.1 months in the phaco-trab group and 12.9 ± 3.9 months in the phaco-KDB group (*P***=** 0.703).

**Figure 1:**
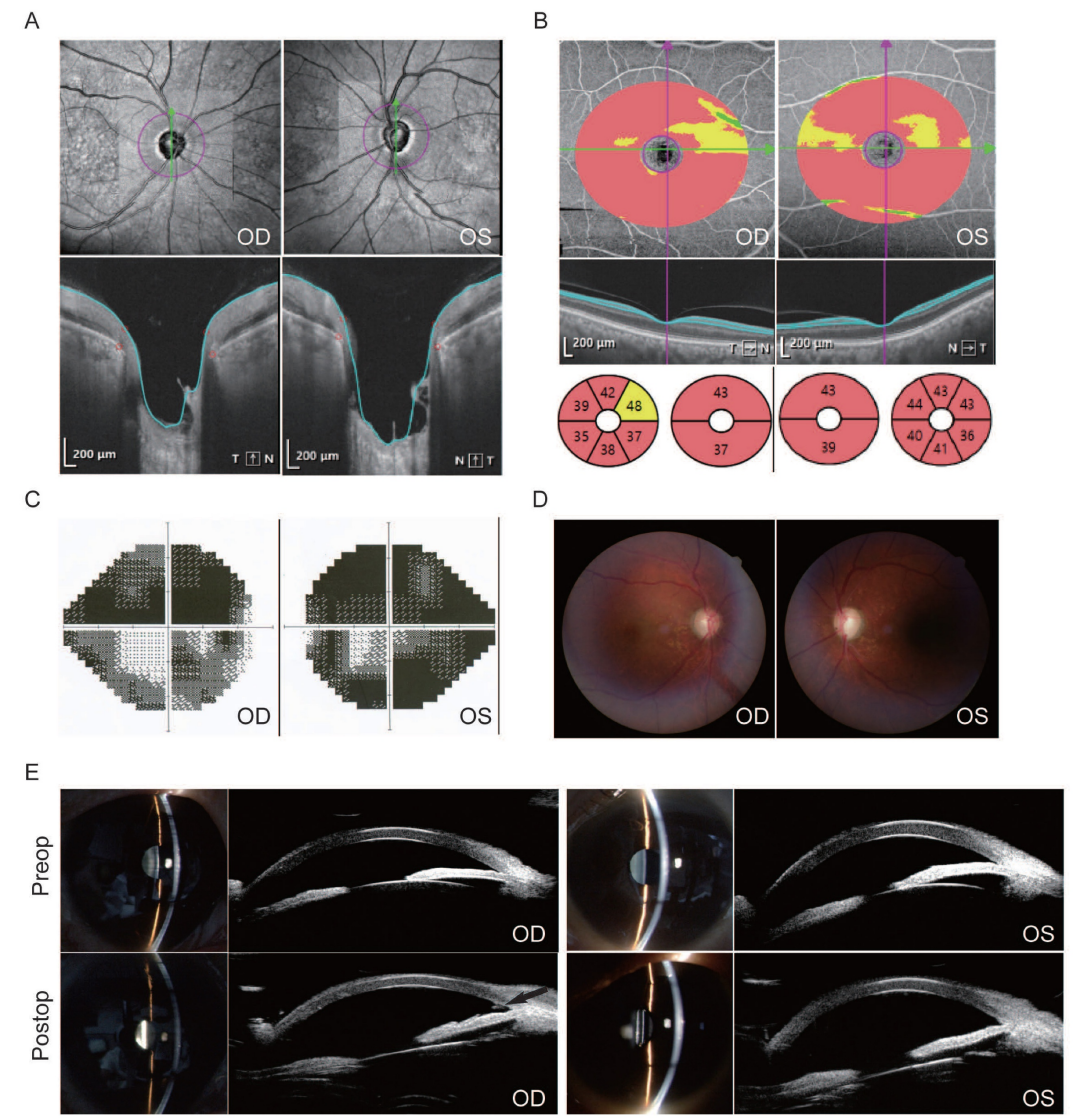
Ocular imaging of a 59-year-old male patient with advanced PACG treated by Phaco-KDB in both eyes. A and B: Optical coherence tomography confirmed an optic disc enlargment and depression, and thinning of retinal ganglion cell complex. C: Humphrey visual field testing demonstrated severe deficits. D: Fundus examination documented a cup-disk ratio (C/D) of 0.8. E: Slit-lamp biomicroscopy of the ocular anterior segment identified shallow anterior chamber. The UBM test confirmed closed anterior chamber angles. After surgery, the wall of the TM was patent at the surgical site, and the residual margin of the excised medial TM wall was observable in the right eye by UMB scanning (black arrow).

Ocular imaging of a representative patient with advanced PACG treated by Phaco-KDB demonstrated characteristic preoperative pathology and successful postoperative anatomical changes (Figure 1). Preoperative evaluation revealed significant glaucomatous damage, including bilateral optic disc cupping with depression and profound thinning of the retinal ganglion cell complex on optical coherence tomography (OCT), severe visual field loss on Humphrey testing, a cup-to-disc ratio of 0.8 on fundus examination, and a shallow anterior chamber with angle closure confirmed by slit-lamp biomicroscopy and ultrasound biomicroscopy (UBM). Postoperative UBM imaging confirmed the efficacy of the procedure, clearly demonstrating a patent internal ostium at the surgical site with the distinct residual margin of the excised medial trabecular meshwork wall (black arrow, Figure 1), indicative of successful restoration of aqueous outflow access.

### Visual Acuity

**Figure 2:**
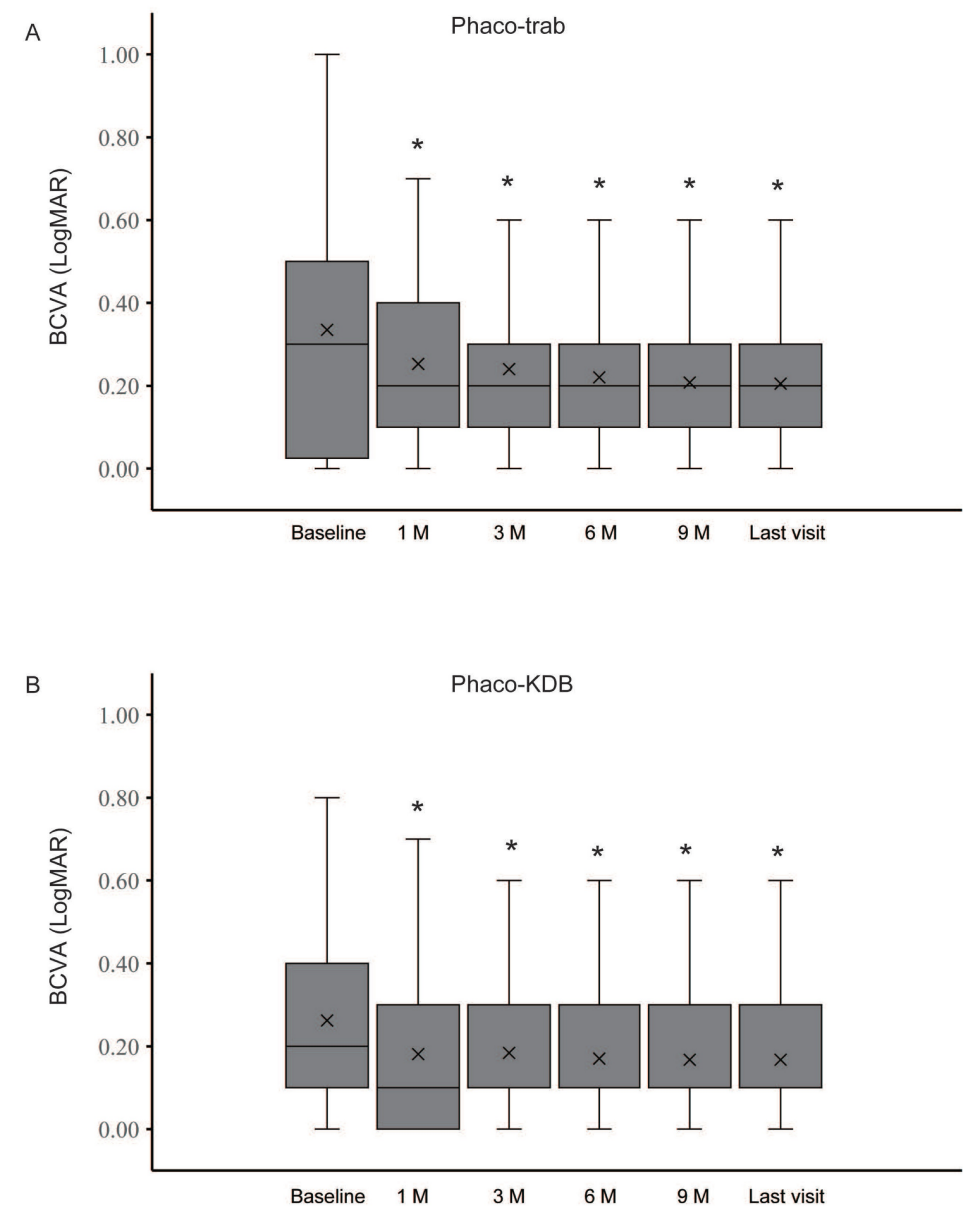
Box-and-whisker plots illustrating baseline and postoperative changes in BCVA at each follow-up visit. A: The horizontal centerline represents the median (50th percentile), while the lower and upper bounds of the box denote the 25th and 75th percentiles, respectively. The upper and lower whiskers extend to the maximum and minimum values within a 1.5× interquartile range (IQR), and cross symbols indicate the mean values. B: At 1 month postoperatively, the lower bound of the box (25th percentile) overlaps with the minimum value. From 3 months postoperatively onward, the median (50th percentile) overlaps with the lower bound of the box (25th percentile). In both the phaco-trab and phaco-KDB groups, postoperative BCVA significantly improved at each follow-up visit (*P*<0.01).

Box-and-whisker plots illustrate the baseline and postoperative changes in BCVA for the phaco-trab and phaco-KDB groups (Figure 2). In both groups, postoperative BCVA improved at each follow-up visit (all *P*<0.01). No statistically significant differences were observed between the two groups at any time point (Table 2). Additionally, a BCVA of 20/40 or better was achieved in 80% (32 eyes) of the phaco-trab group and 81.1% (30 eyes) of the phaco-KDB group.

### Intraocular Pressure and Medications

**Figure 3:**
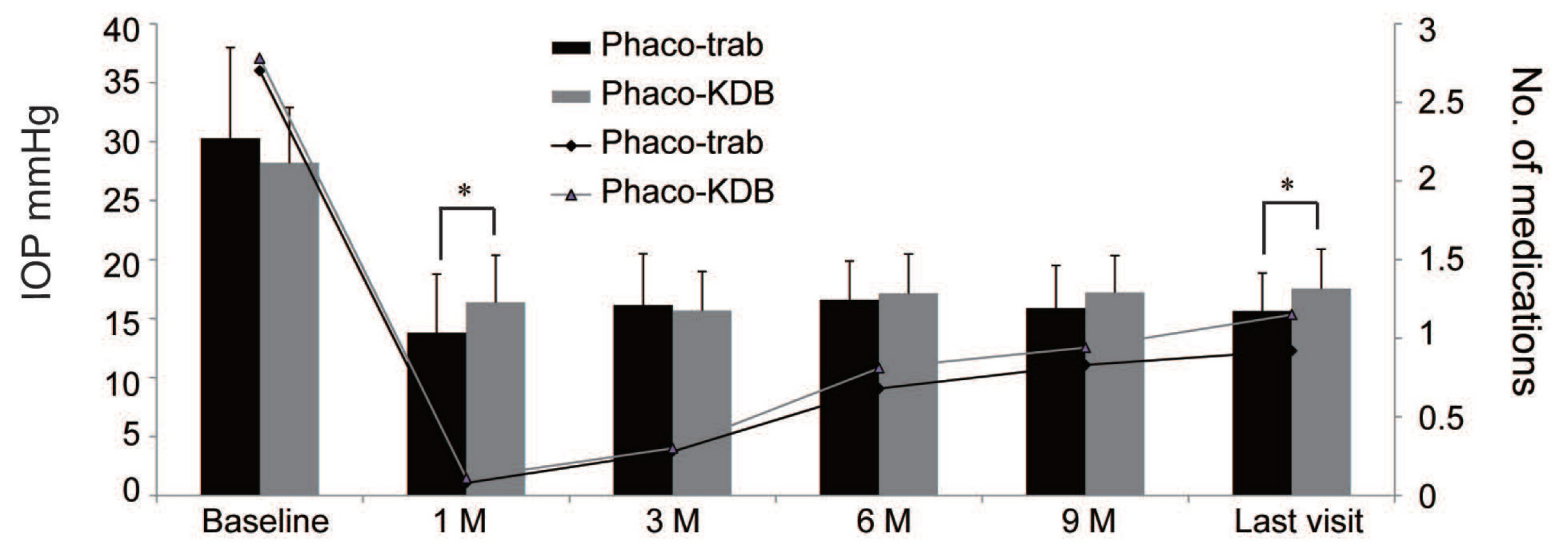
Changes in IOP and number of glaucoma medications in the two groups.

In both groups, significant reductions in IOP and the number of medications were observed at each follow-up visit (Figure 3, Table 3). At baseline, the mean IOP was 30.29 ± 7.69 mmHg in the phaco-trab group and 28.20 ± 4.70 mmHg in the phaco-KDB group. By the last follow-up, the mean IOP had decreased to 15.64 ± 3.22 mmHg (phaco-trab group) and 17.55 ± 3.34 mmHg (phaco-KDB group), respectively. Statistically significant intergroup differences were observed at 1 month postoperatively (*P* = 0.015) and the last follow-up (*P* = 0.041). Preoperatively, the mean number of topical IOP-lowering medications was 2.7 ± 0.76 in the phaco-trab group and 2.78 ± 0.79 in the phaco-KDB group. At the last follow-up, this had declined to 0.92 ± 0.41 (phaco-trab group) and 1.15 ± 0.56 (phaco-KDB group), respectively. Patients in the phaco-KDB group required a few more medications at the last follow-up (*P* = 0.027).

### Success Rate

**Figure 4:**
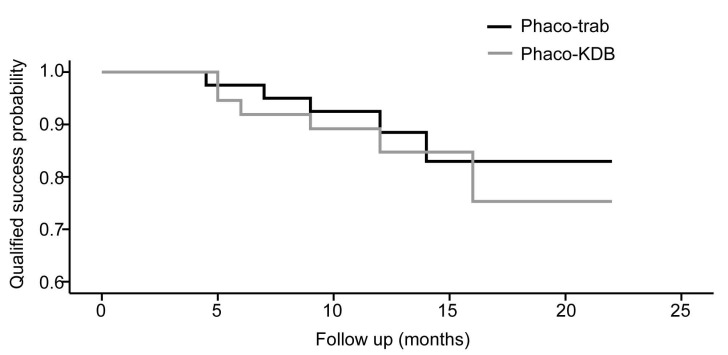
The Kaplan-Meier survival curves for qualified success rate in the two groups.

At the last follow-up, the qualified success rate was 87.5% in the phaco-trab group and 83.8% in the phaco-KDB group. On the basis of the Kaplan-Meier survival analysis, there was no difference in the qualified success probability between the two groups (*P* = 0.604, Figure 4). Regarding complete success rates, the phaco-trab group achieved a rate of 77.5%, while the phaco-KDB group had a rate of 67.6%. In the phaco-KDB group, 3 eyes required adjunctive filtration surgery between 3 and 6 months postoperatively due to uncontrolled IOP.

### Visual Field Defect and Complication

**Figure 5:**
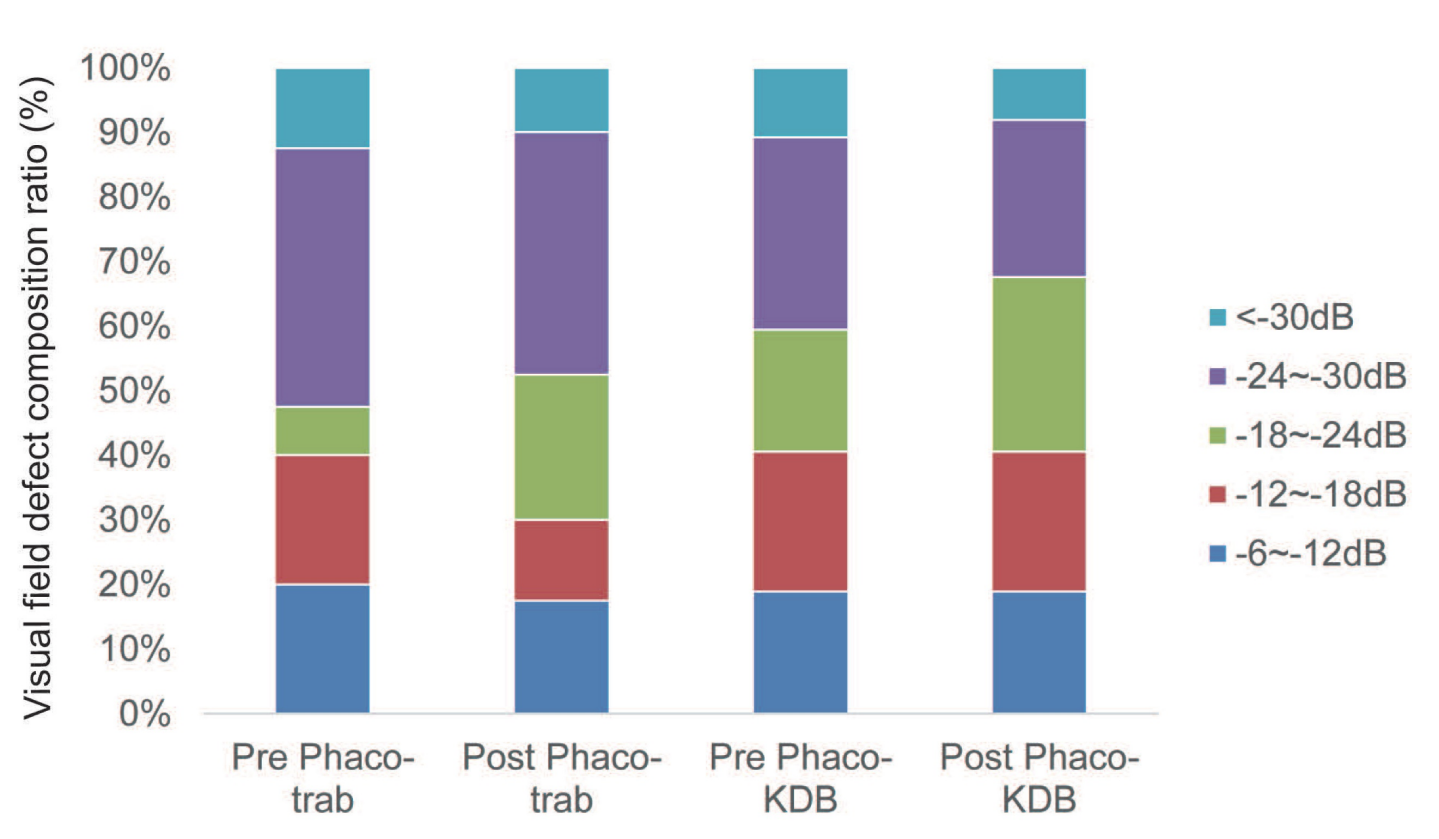
Composition ratio of visual field defect severity in the two groups at baseline and last follow-up. No statistically significant progression of VF defects was observed during the follow-up period (*P*>0.05).

At baseline, advanced VF defects were present in 80% of eyes in the phaco-trab group and 81.1% of eyes in the phaco-KDB group (Table 4). By the last follow-up, no statistically significant progression of VF defect was observed in either group (Figure 5).

In the phaco-KDB group, hyphema was the most common early postoperative finding: it occurred in 25 eyes (67.6%) on the first postoperative day. All hyphema cases were self-limiting and resolved spontaneously within 1-2 weeks. In the phaco-trab group, anterior chamber shallowing was noted in 5 eyes (12.5%) and resolved with medical treatment after 2 weeks. No serious intraoperative or postoperative complications were reported in either group.

## Discussion

PACG affects over 20 million individuals worldwide and is a leading cause of blindness.^11^ Its prevalence is highest in Asia, where it affects 1.09% of individuals aged ≥ 40 years.^12^ Notably, the reported blindness rate in PACG patients is 27.0%, significantly higher than the 8.9% observed in POAG patients.^13^ The pathogenesis of angle closure involves multiple mechanisms, including: pupillary block, plateau iris configuration, lens enlargement, anterior rotation of the ciliary body, choroidal thickness and uveal expansion. Interventions to halt progressive angle closure or reopen the anterior chamber angle, such as laser peripheral iridotomy, iridoplasty, lens extraction, and goniosynechialysis, have demonstrated efficacy.^14^ A landmark randomized controlled trial^15^ has established clear-lens extraction as a first-line treatment for PACG, leading to the increasing adoption of phacoemulsification by surgeons, particularly for patients with primary angle closure (PAC) or mild PACG aged ≥ 40 years.

This study focuses on moderate to severe PACG cases with concurrent cataract. In these patients with a history of LPI, phacoemulsification alone or combined with goniosynechialysis may insufficiently achieve target IOP. A meta-analysis of 14 randomized controlled trials has demonstrated that phaco-trab offers advantages over standalone phacoemulsification for PACG, including superior IOP control and reduced reliance on glaucoma medications. Additionally, phaco-trab has been shown to have a significantly lower failure rate than isolated trabeculectomy in PACG patients over 24 months of follow-up,^16^ establishing its utility as a preferred treatment for medically uncontrolled cases or advanced glaucoma. Economically, phaco-trab also represents a cost-effective strategy for IOP reduction in PACG eyes with cataract.^17^ However, the limitations of filtration bleb-dependent surgeries cannot be entirely circumvented.

Over the past two decades, the dominance of classic trabeculectomy has faced challenges due to the gradual emergence of MIGS, whose advantages in safety and rapid recovery have become increasingly evident. Collectively termed MIGS, these innovative techniques, devices, and implants, such as KDB goniotomy, gonioscopy-assisted transluminal trabeculotomy, Trabectome, iStent, XEN, and endoscopic cyclophotocoagulation, offer distinct benefits over traditional filtration surgery. Multiple studies 18-23 have demonstrated that MIGS procedures combined with phacoemulsification hold promise for PACG patients with cataract, delivering outcomes such as IOP reduction, medication sparing, and VA improvement. A network meta-analysis 24 comparing IOP-lowering efficacy across randomized clinical trials for PAC/PACG has shown that phacoemulsification + goniosynechialysis + goniotomy yield the most promising results for IOP reduction. Notably, the specific benefits of phacoemulsification combined with goniotomy for advanced PACG during 2- year follow-up has been reported recently.^25^

KDB goniotomy offers distinct technical advantages over other MIGS procedures, including a shorter learning curve, no requirement for additional implants, and avoidance of expensive instrumentation. The dual-blade design of the KDB device enables complete resection of the trabecular meshwork (TM) without residual TM leaflets.^26^ Historically, KDB goniotomy has been predominantly used in open angle glaucoma (OAG). Cumulative evidence demonstrates that KDB goniotomy improves surgical outcomes in OAG or ocular hypertension patients, achieving IOP reduction and medication sparing.^27^-^29^ Available data from ACG populations include: a retrospective single-arm study of 42 ACG eyes with cataract showed that phaco-KDB safely reduced IOP and glaucoma medications while improving visual acuity at 12 months.^18^ A 24-month longitudinal analysis of 11 ACG eyes demonstrated about 30% IOP reduction and > 50% medication reduction with phaco-KDB.^30^ In 72 PACG patients, phaco-KDB achieved 65.3% complete success (unmedicated IOP ≤18 mmHg) and 79.2% qualified success over an average follow-up of 11.6±3.7 months.^31^ These findings suggest feasibility and short to medium term efficacy of phaco-KDB in PACG. Until now, whether phaco-KDB has a comparable efficacy as phacotrabeculectomy in moderate to severe PACG has not been fully understood.

In our study, phaco-trab exhibited significantly greater IOP reduction than phaco-KDB at 1 month postoperatively, which indicates that ab externo filtration-based surgery has a greater ability to achieve target IOP in the early postoperative period. At the last follow-up, the mean IOP in the phaco-KDB group (17.55 ± 3.34 mmHg) was higher than that in the phaco-trab group (15.64 ± 3.22 mmHg), with a difference of < 2mmHg. In contrast, previous studies reported a 4 mmHg difference in IOP reduction between phaco-trab and phacogoniotomy at 12 months.^32^ This finding demonstrates that phaco-KDB is non-inferior to phaco-trab in terms of IOP control. Notably, over a 12-month follow-up, phaco-KDB achieved a qualified success rate of 83.8%, which was on par with the 87.5% rate observed in the phaco-trab group. Unlike prior reports, our study cohort included PACG patients with higher baseline IOP and more advanced disease stages—factors that provide greater confidence in exploring the clinical value of phaco-KDB in future applications. While trabeculectomy remains the gold standard for achieving the lowest intraocular pressures, it does so at the cost of a higher complication rate. In contrast, the Phaco-KDB procedure achieves a more favorable balance between therapeutic efficacy and safety, making it particularly suitable for cases where a IOP reduction is sufficient.

Regarding visual acuity outcomes, most patients in both groups experienced improvements in BCVA, accompanied by stable visual field mean deviation. Although there was no significant intergroup difference in mean BCVA at any follow-up visit, eyes in the phaco-KDB group showed a trend toward better visual acuity outcomes. This trend may be attributed to faster visual recovery and a more stable tear film associated with the minimally invasive nature of phaco-KDB.

Nowadays, there are no objective preoperative tests to know whether or not the distal outflow channels are functional, which may make KDB procedure uncertain in IOP-lowing effect. Imaging technologies like OCT angiography or more sophisticated perfusion tests that might one day allow us to objectively "see" the functionality of the distal outflow system preoperatively. In the absence of a definitive test for distal outflow function, the surgeon must make an educated "guess". This guess is based on glaucoma type, severity, conjunctival status, and the patient's individual needs and risk tolerance. KDB is an excellent, safe option for the right patient. For the patient with severe, long-standing PACG in our study where distal outflow failure is a real concern, after phacoemulsification we circumferentially separate PAS to re-open the trabecular meshwork followed by a 3-4 clock-hour incision of Schlemm's canal. The surgeon and patient must accept that it might not be sufficient, additional eye drops or trabeculectomy may still be needed later. Since trabeculectomy remains the gold standard for achieving low target pressures, accepting its higher risk profile, with consideration of the late stage glaucoma particularly.

The follow-up protocol differs significantly between phaco-KDB and phaco-trab because the procedures have fundamentally different risk profiles and timelines for success and failure. For phaco-KDB, The post-operative course for KDB is typically benign and resembles an uncomplicated cataract surgery recovery, but with a crucial emphasis on monitoring the anterior angle. During first 2 weeks, the main concern is a transient hyphema from the goniotomy site, which usually resolves spontaneously. Intermediate phase (1-3 months) is the critical period for judging the IOP-lowering effect by gonioscopy to assess the PAS or fibrosis. After 6 months, the primary goal is to manage a stable IOP and monitor glaucoma progression. For phaco-trab, the follow-up is a long-term, active process of bleb management. When a trabeculectomy is needed after phaco-KDB, the conjunctival status is a major advantage, making it a logical next step in the surgical treatment algorithm for glaucoma. The anterior chamber is also often more stable post-cataract surgery. The initial phaco-KDB was a lower-risk procedure that may have provided several years of adequate IOP control, delaying the need for the higher-risk trabeculectomy.

This single-center, non-randomized retrospective study evaluated patients with moderate-to-severe PACG and concurrent cataract, with a mean follow-up duration of over 12 months. When interpreting these results for this specific patient cohort, several critical limitations must be acknowledged: the study design; selection bias; the relatively small sample size and limited follow-up period. The inclusion of both eyes from certain patients in the analysis may have introduced inter-eye correlation bias, which could influence the results. Therefore, future studies would benefit from a more robust design that enrolls only one eye per subject to ensure the statistical integrity of the findings. Additionally, variations in the type of microhook used across different studies and surgeon variability in technical skills may have influenced surgical outcomes.

In conclusion, phaco-KDB is an effective *ab interno* procedure that achieves comparable IOP control and visual acuity improvement to phaco-trab in patients with moderate-to-severe PACG and concurrent cataract. Notably, phaco-KDB offers superior safety profiles and faster visual rehabilitation, while reducing the need for invasive filtration surgery. For cases of moderate-to-severe PACG, phaco-KDB may serve as a viable alternative to delay or abviate the need for traditional trabeculectomy and represents an important addition to the surgical armamentarium-one that provides patients with a distinct therapeutic benefit profile.

## Figures and Tables

**Table 1:** Demographic characteristics and baseline clinical parameters of patients with PACG

Parameter	Phaco-trab group Mean ± SD (range)	Phaco-KDB group Mean ± SD (range)	*P* value
Eye (n)	40	37	
Gender (F/M)	25/14	22/13	0.912^†^
Age (y)	61.40±9.19 (46-84)	63.76±6.97 (47-79)	0.212
BCVA (log MAR)	0.31±0.36 (0-2.0)	0.30±0.40 (0-2.0)	0.901
IOP (mmHg)	30.29±7.69 (23-51)	28.20±4.70 (22-45)	0.155
Medications (n)	2.70±0.76(2-4)	2.78±0.79 (2-4)	0.635
ACD (mm)	2.17±0.42 (1.40-2.80)	2.29±0.40 (1.41-3.24)	0.199
AL (mm)	22.35±0.63(20.68-23.94)	22.63±0.81(20.78-24.7)	0.095
LT (mm)	4.79±0.35 (4.00-5.24)	4.91±0.41 (4.21-5.77)	0.148
IOL power (D)	23.51±2.07 (19.5-30.0)	24.0±2.70 (19.5-31.5)	0.339
PAS (°) ≥ 180° < 180°	279.00±89.67 (90-360) 32 (80%) 8 (20%)	240.81±92.06 (90-360) 31 (83.8%) 6 (16.2%)	0.168^#^
VF defect (dB) Moderate (-12 < MD ≤ -6 dB) Advanced (MD ≤ -12 dB) Months of follow-up	-21.65±7.95 (-7.39 to -30.77) 8 (20%) 32 (80%) 13.5±4.1	-20.48±7.82 (-6.14 to -32) 7 (18.9%) 30 (81.1%) 12.9±3.9	0.518^#^ 0.703

BCVA: best corrected visual acuity, logMAR: logarithm of the minimum angle of resolution, IOP: intraocularpressure, ACD: anterior chamber depth, AL: axial length, LT: lens thickness, IOL: intraocular lens, PAS: peripheral anterior synechiae, VF: visual field, MD: mean deviation, SD: standard deviation. *P*: result of t-test, # Mann-Whitney U test or † Chi-square test.

**Table 2:** Baseline and postoperative BCVA of patients with PACG in the two treatment groups

BCVA (log MAR)	Phaco-trab group (Mean ± SD)	Phaco-KDB group (Mean ± SD)	*P* value
Baseline	0.34±0.32	0.30±0.40	0.268
1 month	0.25±0.19	0.18±0.20	0.110
3 months	0.24±0.16	0.18±0.16	0.124
6 months	0.22±0.16	0.17±0.16	0.170
9 months	0.21±0.17	0.17±0.15	0.276
Last visit	0.21±0.16	0.17±0.16	0.298
*P* value	<0.01*	<0.01*	

* At each follow-up, the postoperative BCVA is better than that of the baseline.

**Table 3:** Baseline and postoperative IOP and number of glaucoma medications in the two treatment groups

IOP (mmHg) Medication (n)	Phaco-trab group (Mean ± SD)	Phaco-KDB group (Mean ± SD)	*P* value
Baseline	30.29±7.69 2.7±0.76	28.20±4.70 2.78±0.79	0.155 0.635
1 month	13.79±4.97 0.08±0.27	16.37±4.00 0.11±0.24	0.015* 0.867
3 months	16.13±4.37 0.28±0.55	15.68±3.32 0.30±0.91	0.615 0.316
6 months	16.60±3.28 0.68±0.83	17.14±3.33 0.81±0.89	0.480 0.210
9 months	15.87±3.63 0.83±0.15	17.21±3.12 0.94±0.24	0.269 0.361
Last visit	15.64±3.22 0.92±0.41	17.55±3.34 1.15±0.56	0.041* 0.027*
*P* value^#^	<0.01 <0.01	<0.01 <0.01	

*: Statistically significant intergroup differences in IOP are observed at 1 month and the last visit, and statistically significant difference in medication is observed at the last visit. (*P*< 0.05) #: At each follow-up, the IOP and the number of medications is lower than the baseline.

**Table 4:** Baseline and postoperative visual field defect severity in the two treatment groups

VF defect (dB)	Phaco-trab group (Mean ± SD)	Phaco-KDB group (Mean ± SD)	*P* value
Baseline	-21.65±7.95 (-7.39 to -30.77)	-20.48±7.82 (-6.14 to -32)	0.518
Last visit	-21.49±7.73 (-6.65 to -31.67)	-19.83±7.34 (-6.37 to -31.77)	0.338
*P* value	0.534	0.064	

No statistically significant difference is observed.

## Data Availability

Datasets from the current study are not publicly available due to compliance with patient privacy. Summary statistics are available from the corresponding author on reasonable request.

## Abbreviations

MIGS: Minimally invasive glaucoma surgery
PACG: Primary angle closure glaucoma
POAG: primary open angle glaucoma
KDB: Kahook Dual Blade
VF: Visual field
IOP: Intraocular pressure
BCVA: Best-corrected visual acuity
Log MAR: Logarithm of the minimum angle of resolution
MD: Mean deviation
AL: Axial length
LT: Lens thickness
PAS: Peripheral anterior synechiae
LPI: laser peripheral iridotomy
RNFL: Retinal nerve fiber layer
ACD: Anterior chamber depth
TM: Trabecular meshwork
SD: Standard deviation
OCT: optical coherence tomography
UBM: ultrasound biomicroscopy
